# SARS-CoV-2 ORF3a drives dynamic dense body formation for optimal viral infectivity

**DOI:** 10.21203/rs.3.rs-4292014/v1

**Published:** 2024-05-17

**Authors:** Stella Hartmann, Lisa Radochonski, Chengjin Ye, Luis Martinez-Sobrido, Jueqi Chen

**Affiliations:** 1 Department of Microbiology, University of Chicago, Chicago, IL, USA 60637; 2 Howard Taylor Ricketts Laboratory, University of Chicago, Lemont, IL, USA 60439; 3 Texas Biomedical Research Institute, San Antonio, TX, USA 78227

## Abstract

SARS-CoV-2 uses the double-membrane vesicles as replication organelles. However, how virion assembly occurs has not been fully understood. Here we identified a SARS-CoV-2-driven membrane structure named the 3a dense body (3DB). 3DBs have unusual electron-dense and dynamic inner structures, and their formation is driven by the accessory protein ORF3a via hijacking a specific subset of the *trans*-Golgi network (TGN) and early endosomal membranes. 3DB formation is conserved in related bat and pangolin coronaviruses yet lost during the evolution to SARS-CoV. 3DBs recruit the viral structural proteins spike (S) and membrane (M) and undergo dynamic fusion/fission to facilitate efficient virion assembly. A recombinant SARS-CoV-2 virus with an ORF3a mutant specifically defective in 3DB formation showed dramatically reduced infectivity for both extracellular and cell-associated virions. Our study uncovers the crucial role of 3DB in optimal SARS-CoV-2 infectivity and highlights its potential as a target for COVID-19 prophylactics and therapeutics.

## INTRODUCTION

Severe acute respiratory syndrome coronavirus-2 (SARS-CoV-2) is a positive-sense single-stranded RNA virus that causes the coronavirus disease 2019 (COVID-19). To date, more than 700 million cases of COVID-19 have been reported, resulting in more than 7 million reported deaths^1^. SARS-CoV-2 is genetically similar to the previously discovered SARS-CoV responsible for the 2002–2003 SARS outbreak^2,3^. SARS-CoV and SARS-CoV-2 both form interconnected double-membrane vesicles (DMVs) derived from the endoplasmic reticulum (ER) to serve as replication organelles where viral RNA replication occurs^4–9^. In addition, ERGIC-derived structures were proposed to be important for the assembly of mature SARS-CoV-2 virions^10^. The Golgi apparatus, mitochondria, and peroxisomes were also proposed to be remodeled by SARS-CoV-2^4^. However, how these membrane structures coordinate to orchestrate the virion assembly, and whether there are differences in membrane remodeling driven by SARS-CoV-2 and SARS-CoV, have not been fully characterized.

Here we identified a membrane structure assembled during SARS-CoV-2 infection, which we termed the 3a dense body (3DB). 3DBs are giant electron-dense spherical structures with dynamic inner structures. Their formation is driven by the accessory protein open reading frame (ORF)3a from SARS-CoV-2 (hereinafter referred to as 3a^CoV2^). 3a^CoV2^ has been proposed to be a viral small ion channel protein (viroporin)^11,12^, although its ion channel activity has remained controversial in recent studies^13,14^. Our previous study has shown that 3a^CoV2^ is the most important accessory protein in SARS-CoV-2 virulence in a K18-hACE2 transgenic mouse model of infection^15^. A mutant virus deficient in 3a^CoV2^ (Δ3a) exhibited the highest improvement in lung pathology and survival compared to those infected with wild type (WT) or mutant viruses deficient in other accessory ORF proteins^15^. Reduced virulence in animals correlated with a defect in Δ3a viral transmission as indicated by reduced plaque size^15^. However, the molecular and cellular mechanisms underlying the critical roles of 3a^CoV2^ as a virulence factor have remained unclear, although recent studies have proposed its involvement in lysosomal exocytosis-mediated viral egress, autophagy, and late endosome/lysosome trafficking^13,16–25^. Our discovery unveils a previously uncharacterized function of 3a^CoV2^ to assemble 3DBs via remodeling a specific subset of the host *trans*-Golgi network and early endosomal membrane. 3DBs did not contain other organelle markers including those from the ER, ERGIC, *cis*-Golgi, late endosomes, lysosomes, or autophagosomes, suggesting that 3DBs are distinct from other well-known SARS-CoV-2-associated structures, and that 3DB formation is independent of the previously characterized roles of ORF3a in modulating lysosome function and autophagy.

A number of mammalian coronaviruses share similar genomic sequences with SARS-CoV and SARS-CoV-2. Together, these viruses form the group of SARS-related coronaviruses (SARSr-CoVs)^10,26^. ORF3a is conserved among SARSr-CoVs, but not with other human coronaviruses (HCoVs) such as Middle East respiratory syndrome coronavirus (MERS-CoV), HCoV-NL63, or HCoV-229E^10,27^. Interestingly, although 3DB formation activity is conserved among ORF3a from related bat and pangolin coronaviruses, it was lost in the homologs from the closely related SARS-CoV (3a^CoV1^) and a civet coronavirus proposed to be an intermediate species for SARS-CoV, highlighting an unexpected functional divergence in ORF3a during evolution. Using extensive domain swapping and bioinformatic analysis, we have identified seven key amino acid residues crucial for the 3DB formation activity. WT recombinant SARS-CoV-2 drove the formation of 3DBs, which then recruit the viral structural proteins spike (S) and membrane (M). In contrast, an engineered SARS-CoV-2 with the seven key residues of 3a^CoV2^ mutated, completely lost the ability to form 3DBs and thus was unable to recruit S and M to the dense bodies. This mutant virus had significant reduced infectivity for both extracellular and cell-associated virions, suggesting that 3DBs facilitate the trafficking of S and M for virion assembly to achieve maximal infectivity. Our findings uncovered a unique and evolutionarily conserved membrane reorganization activity and its role in the viral life cycle of SARS-CoV-2. The reduced pathogenicity of SARS-CoV-2 containing mutations in ORF3a highlights the potential of targeting ORF3a for the rational development of live-attenuated vaccines to combat SARS-CoV-2 and future emerging HCoVs given the highly conserved nature of this remodeling activity in bat progenitor coronaviruses. Screening of inhibitors targeting 3a^CoV2^-mediated 3DB formation, such as those that bind to or modify the seven key residues, may provide promising directions for the discoveries of innovative COVID-19 therapeutics.

## RESULTS

### Identification of 3a^CoV2^-driven dense bodies

**Figure 1:**
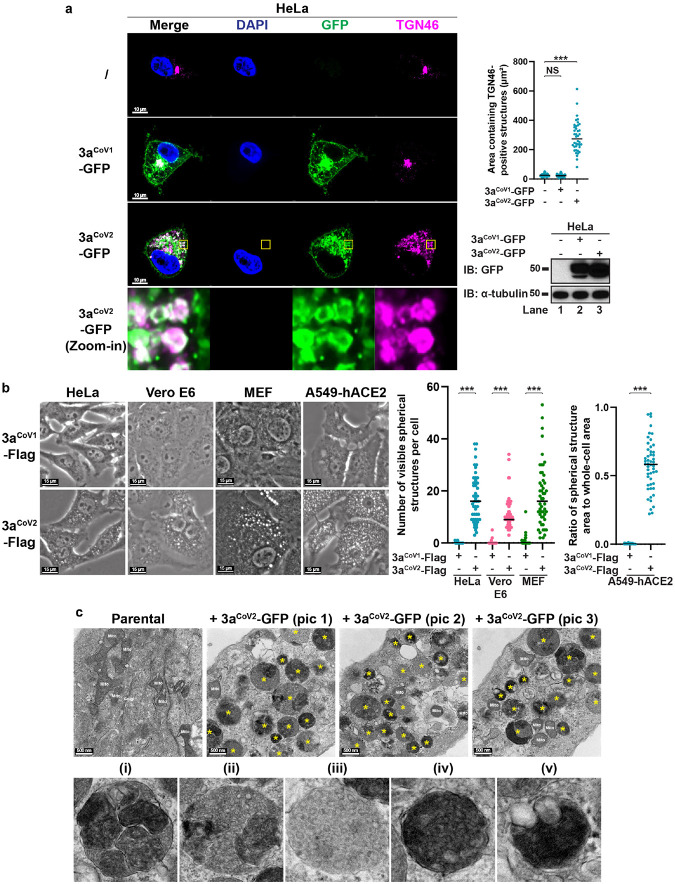
3aCoV2 but not 3aCoV1 induces giant dynamic dense bodies. (a) HeLa cells stably expressing 3aCoV1-GFP or 3aCoV2-GFP were immunostained for TGN46, a TGN marker. The parental HeLa cells (‘/’) were used as control. A magnified region of HeLa 3aCoV2-GFP cells highlighted the spherical structures positive with 3aCoV2-GFP and TGN46. Areas containing TGN46-positive structures were measured with ImageJ (n = 40 cells/sample; mean ± s.d.; two-sided t-test; ***, p<0.001; NS, not significant; black line indicates median value). GFP immunoblotting was performed to indicate that the 3a-GFP proteins were expressed at comparable levels. Representative data from at least three independent experiments are shown. (b) The indicated cell lines stably expressing 3aCoV1-Flag or 3aCoV2-Flag were imaged with phase contrast microscopy. For HeLa, Vero E6, and MEF series, the number of spherical structures per cell (visible on the current focal plane with clear DAPI signal) was quantified. For A549-hACE2 series, because the massive formation of spherical structures made it challenging to quantify the spherical structure number, we measured the ratio of spherical structure area to whole-cell area instead. n = 40 cells/sample; mean ± s.d.; two-sided t-test; ***, p<0.001; black line indicates median value. Representative data from at least three independent experiments are shown. (c) Upper panel: Vero E6 parental cells or cells stably expressing 3aCoV2-GFP were imaged with transmission electron microscopy (TEM). Three pictures (pic 1–3) are shown to highlight different morphologies of 3a dense bodies (3DBs) labeled with *. Mito, mitochondria. Lower panel: five subtypes of 3DBs based on their morphological features are shown: (i) consisting of several membranous sub-compartments; (ii) consisting of dense pebble-like substructures and membranous sub-compartments; (iii) consisting of dense pebble-like substructures; (iv) highly electron-dense structures; (v) similar to (iv), but fused to one or multiple electron-lucent vesicle-like structures. Representative images from >40 cells per condition are shown.

The *trans*-Golgi network (TGN) serves as the major sorting compartment and the center for terminal processing and modifications of newly synthesized proteins^28^. Our previous work discovered that several microbial factors, including the bacterial ionophore nigericin, induce TGN disassembly into vesicles without dispersing the *cis*/medial-Golgi or other organelles^29^. The dispersed TGN vesicles then serves as a signaling platform for the assembly and activation of the NLRP3 inflammasome^29^. This indicates that the TGN can be specifically remodeled through host-bacteria interactions. However, whether viral proteins possess similar remodeling activity remains unknown. We hypothesized that viroporins might be potential TGN-remodeling factors. We selected a group of viroporins derived from phylogenetically diverse groups of DNA and RNA viruses (**Extended Data Fig. 1a**). To prevent the interference of other viral factors, we developed an individual expression system, in which the viroporin genes were cloned into a lentiviral vector for stable expression in HeLa cells. Either N-terminal or C-terminal tagging was used based on previous literature or our pilot experiments to ensure optimal expression and localizations of viroporins. The cells were then fixed and immunostained for TGN46 (also known as TGOLN2 or TGN38), a marker for TGN. We defined TGN remodeling as ≥ 3-fold increase in the average surface area containing TGN46-positive structures with p value < 0.01 (Student’s t-test) compared to that of the parental HeLa cells. Out of the ten viroporins that were successfully expressed, seven (2B from poliovirus, M2 from influenza A virus, NSP4 from rotavirus, VP4 from human rhinovirus, Vpu from HIV, 3a^CoV1^, and 3a^CoV2^) showed at least partial colocalization with TGN46 (Fig. 1a and **Extended Data Fig. 1b**). However, only 3a^CoV2^ induced dramatic dispersion of TGN46-positive structures from an intact cluster into multiple spherical structures (Fig. 1a, 0.60 ± 0.22 μm in diameter), as quantified by a ~10-fold increase in TGN46-positive area (287.1 ± 105.2 μm^2^ vs. 26.4 ± 9.4 μm^2^). 3a^CoV2^ was mainly localized on these TGN46-postive spherical structures (Fig. 1a, Zoom-in) besides additional cytosolic aggregate and plasma membrane (PM) localization. The ability of 3a^CoV2^ to induce TGN46-positive spherical structures is highly efficient, with 100% penetrance in the stable cell line. Surprisingly, this remodeling activity was not observed for 3a^CoV1^, which was expressed at a comparable level based on immunoblotting (Fig. 1a). 3a^CoV1^ was predominantly localized on the intact TGN cluster besides additional cytosolic aggregate and PM localization (Fig. 1a). For both 3a^CoV2^ and 3a^CoV1^, only the C-terminal but not the N-terminal tagging could be detected via immunostaining (**Extended Data Fig. 2a–c**). The N-terminal tagging did not affect the expression of both 3a proteins or the remodeling ability of 3a^CoV2^ (**Extended Data Fig. 2b–c**), suggesting that the N-terminus of 3a^CoV1^ and 3a^CoV2^ is likely processed during or after translation. We therefore used C-terminally tagged ORF3a for the rest of this study.

The different effects of 3a^CoV2^ and 3a^CoV1^ on TGN46-positive structures were recapitulated in a variety of cell lines, including two that are routinely used for SARS-CoV-2 infection studies^30,31^: (1) Vero E6 (**Extended Data Fig. 3a**), an African green monkey kidney epithelial cell line; (2) A549-hACE2 (**Extended Data Fig. 3b**), a human lung epithelial cell line stably expressing human angiotensin-converting enzyme 2 (hACE2), the receptor for SARS-CoV-2^3^. 3a^CoV2^-induced spherical structures can be detected with phase contrast microscopy in a variety of human, monkey, and mouse cell lines, with visible number ranging from ~20 to a few hundred per cell (Fig. 1b).

To confirm that the remodeling was not caused by overloading the TGN with overexpressed 3a^CoV2^, we established a series of A549-hACE2 cell lines stably expressing 3a^CoV1^-GFP or 3a^CoV2^-GFP at different levels through lentiviral titrations. Strikingly, even at much lower expression level than 3a^CoV1^-GFP, 3a^CoV2^-GFP still potently induced massive spherical structure formation (**Extended Data Fig. 3c**). These results indicate that even low amount of 3a^CoV2^ is sufficient to promote robust remodeling. We also observed that cells expressing 3a^CoV1^ or 3a^CoV2^ were morphologically healthy and could be maintained as stable cell lines for at least two months, indicating that the remodeling does not affect the basal cell survival.

Surprisingly, when imaged with transmission electron microscopy (TEM), 3a^CoV2-^induced structures appeared as giant spherical electron-dense bodies with highly dynamic inner compositions (Fig. 1c, upper panel). These structures can be grouped into five subtypes based on their morphological features (Fig. 1c, lower panel): (i) consisting of several membranous sub-compartments; (ii) consisting of dense pebble-like substructures and membranous sub-compartments; (iii) consisting of dense pebble-like substructures; (iv) highly electron-dense structures; (v) similar to (iv), but fused to one or multiple electron-lucent vesicle-like structures. These five subtypes likely represent different maturation stages and/or different sections of the structures. While all five subtypes were observed at high frequencies, (i) and (ii) were the most abundant ones, suggesting that they may be the mature or most stable forms (see Discussion). These structures are distinct from nigericin-induced TGN vesicles or SARS-CoV/SARS-CoV-2-induced DMVs, with the latter two appearing as electron-lucent vesicles^4,29^ (**Extended Data Fig. 3d**). They are also dramatically different from multivesicular bodies (MVBs)^32^ (**Extended Data Fig. 3d**), lipid droplets^33^ (**Extended Data Fig. 3d**), autophagosomes and related structures^34^, endosomes^32^, and lysosomes^32^. Besides Vero E6 in Fig. 1c, similar 3a^CoV2^-driven structures were also observed in HeLa cells (**Extended Data Fig. 3e**). Given the unusual dense nature of their inner compositions, we named these structures the 3a dense bodies (3DBs).

### 3a^CoV2^ specifically remodels a subset of TGN membrane

**Figure 2:**
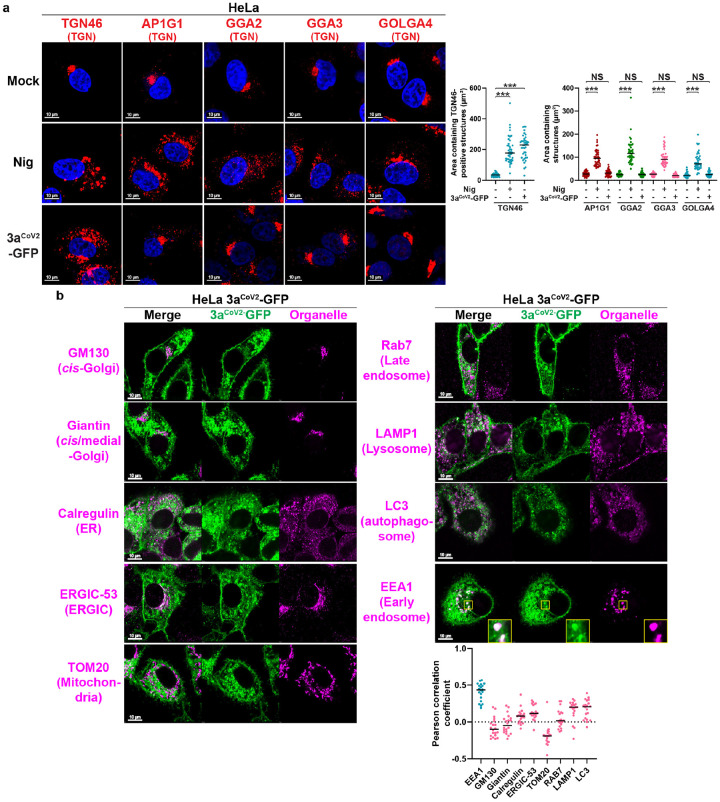
3aCoV2 hijacks a specific subset of TGN and early endosomal membranes to form 3DBs. (a) HeLa cells were treated with 1.5 μL/mL DMSO solvent (‘Mock’) or nigericin (Nig, 10 μM = 1.5 μL/mL dissolved in DMSO) for 80 minutes (min). Together with HeLa cells stably expressing 3aCoV2-GFP, these cells were immunostained for the indicated TGN markers. Areas containing TGN-marker-positive structures were measured with ImageJ (n = 40 cells/sample; mean ± s.d.; two-sided t-test; ***, p<0.001; NS, not significant; black line indicates median value). Representative data from at least three independent experiments are shown. (b) HeLa cells stably expressing 3aCoV2-GFP were immunostained for the indicated organelle markers. For EEA1 immunostaining, a magnified region highlighted localization of EEA1 on a subset of 3DBs. Colocalization of 3DBs with different organelle markers were quantified with Pearson correlation coefficient using Coloc 2 plugin of ImageJ (n = 20 cells/sample; threshold regression: Costes). Organelle makers with strong and weak colocalization with 3DBs are labeled in blue and pink, respectively). Representative data from at least three independent experiments are shown.

Besides the dramatic difference in TEM morphology, 3DBs and nigericin-induced TGN vesicles also differ in number and diameter (**Extended Data Fig. 4a**), thus raising the question as to whether these two remodeling events are of different nature. We previously discovered that several NLRP3 inflammasome stimuli including nigericin disperse the entire TGN into vesicle structures as indicated by multiple TGN markers^29^. After that, the negatively-charged phospholipid PtdIns4P on the dispersed TGN binds to a polybasic region on NLRP3 to mediate NLRP3 recruitment and inflammasome complex assembly^29^. Interestingly, while nigericin treatment triggered the dispersion of all five TGN markers tested, 3a^CoV2^ only dispersed TGN46 (Fig. 2a). 3a^CoV2^ also failed to disperse PtdIns4P-positive TGN structures as detected by the PtdIns4P-binding protein OSBP^PH^-GFP^35^ (**Extended Data Fig. 4b**). Consistent with our previous finding that the dispersed PtdIns4P-positive TGN structures are required for NLRP3 activation^29^, 3a^CoV2^ did not promote NLRP3 puncta formation (**Extended Data Fig. 4c**) or caspase-1 cleavage (**Extended Data Fig. 4d**), two hallmarks of NLRP3 inflammasome activation. 3a^CoV2^ did not prevent nigericin-induced formation of bigger TGN46 vesicles (**Extended Data Fig. 4a**) or NLRP3 inflammasome activation (**Extended Data Fig. 4c–d**), suggesting that 3a^CoV2^-mediated TGN remodeling does not interfere with inflammasome-related TGN remodeling. To examine whether 3a^CoV2^ activates the NLRP3 inflammasome during viral infection, we established a RAW 264.7 murine macrophage cell line stably expressing hACE2-Flag and ASC, the adaptor protein downstream of NLRP3. RAW 264.7 cells express endogenous NLRP3 but not ASC^36,37^, and therefore exogenous expression of ASC in this cell line is often used to reconstitute the inflammasome pathway^29,38^. The expression of hACE2-Flag allows this cell line to be infected with SARS-CoV-2. As expected, nigericin treatment resulted in dramatic formation of ASC specks (**Extended Data Fig. 4e**), a hallmark of inflammasome activation^39,40^. In contrast, cells infected with SARS-CoV-2 (USA-WA1) had a minimal level of ASC speck formation (**Extended Data Fig. 4e**) and no detectable caspase-1 or IL-1β cleavage (data not shown). Our data indicate that 3a^CoV2^ remodels the TGN in a manner distinct from previously characterized NLRP3 inflammasome stimuli, and as a result, is not a potent NLRP3 stimulus either expressed alone or during viral infection.

To examine whether 3a^CoV2^ hijacks membranes from other organelles, we imaged a series of organelle markers. 3a^CoV2^ did not disperse the *cis*- or medial-Golgi (Fig. 2b), again highlighting its specificity. In addition, 3DBs did not contain organelle markers GM130 (*cis*-Golgi), giantin (*cis*/medial-Golgi), calregulin (ER), ERGIC-53 (ERGIC), TOM20 (mitochondria), Rab7 (late endosome), LAMP1 (lysosome), or LC3 (autophagosome) (Fig. 2b). These results indicate that 3DB formation is a previously uncharacterized function of 3a^CoV2^, distinct from its known ability to regulate late endosome/lysosome trafficking and autophagy^13,16–25^. Interestingly, EEA1, an early endosome marker, was recruited to a subset of 3a^CoV2^ structures (Fig. 2b). Our results indicate that 3a^CoV2^ hijacks a specific subset of TGN and early endosomal membranes either directly from these organelles, or indirectly through the cargo exchange between the TGN and early endosomes (see Discussion).

### The C-terminal region of 3a^CoV2^ is critical for 3DB formation

**Figure 3:**
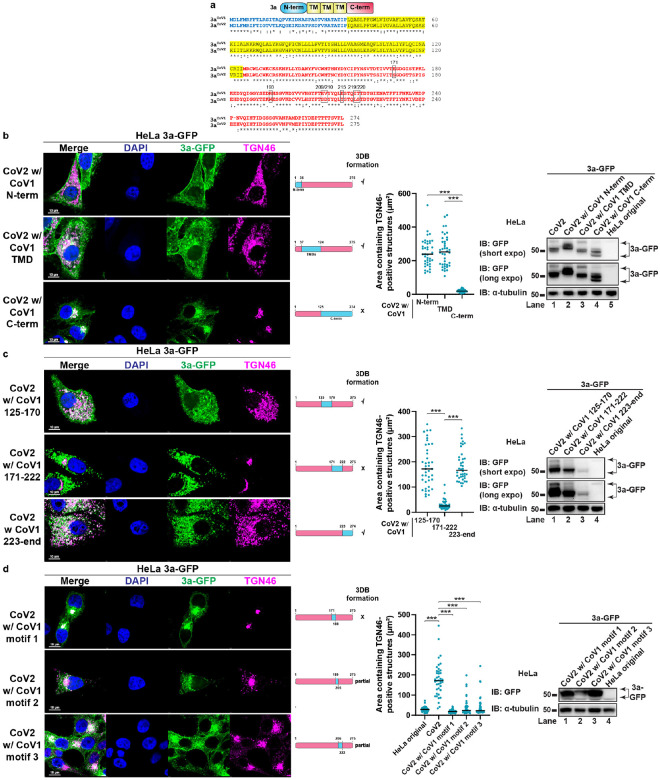
Identification of ORF3a motifs critical for 3DB formation. (a) Alignment of 3aCoV1 and 3aCoV2 protein sequences with Clustal Omega. N-term, TMD and C-term are labeled in blue, yellow, and red, respectively. The seven key residues are highlighted in black frames. (b–d) HeLa cells stably expressing the indicated 3a swapping mutants were immunostained for TGN46. The blue and pink bars represent sequences derived from 3aCoV1 and 3aCoV2, respectively. Areas containing TGN46-positive structures were measured with ImageJ (n = 40 cells/sample; mean ± s.d.; two-sided t-test; ***, p<0.001; black line indicates median value). GFP immunoblotting was performed to compare the 3a-GFP protein levels. Representative data from at least three independent experiments are shown.

3a^CoV2^ and 3a^CoV1^ share a similar domain structure: an N-terminal region (N-term), a transmembrane-domain region (TMD) and a C-terminal region (C-term)^14,41^, with ~72% amino acid (aa) identity (Fig. 3a). We performed a series of domain swapping to identify the region critical for 3DB formation. Replacing N-term (aa 1–36) or TMD (aa 37–124) of 3a^CoV2^ with the corresponding regions in 3a^CoV1^ did not affect 3DB formation (Fig. 3b). In contrast, replacing C-term (aa 125–end) completely abolished the activity, while still maintaining comparable expression level and strong colocalization with the TGN similar to 3a^CoV1^ (Fig. 3b). Consistently, swapping C-term of 3a^CoV1^ with that of 3a^CoV2^ promoted 3DB formation comparable to that caused by 3a^CoV2^ (**Extended Data Fig. 5a**). These data indicate that the C-term of 3a^CoV2^ is crucial for the remodeling activity.

To further narrow down the key region, we divided the C-term into three smaller regions. Swapping aa 171–222 in 3a^CoV2^ completely abolished 3DB formation (Fig. 3c), while swapping the corresponding region in 3a^CoV1^ restored the activity (**Extended Data Fig. 5b**). Swapping the other two smaller regions in the C-term (aa 125–170 and aa 223–end) of 3a^CoV2^ did not affect 3DB formation (Fig. 3c), despite one of them (aa 223–end) being expressed at a much lower level than the other mutants (Fig. 3c, immunoblotting). This is consistent with our observation that 3a^CoV2^ is capable of robust remodeling even at low expression. Consistently, swapping aa 125–170 or aa 223–end in 3a^CoV1^ failed to restore the activity (**Extended Data Fig. 5b**). These results indicate that aa 171–222 of 3a^CoV2^ is crucial for 3DB formation.

We further dissected aa 171–222 into three regions with lengths of 17–18 aa, referred to as motif 1 (aa 171–188), motif 2 (aa 189–205), and motif 3 (aa 206–222). Swapping motif 1 in 3a^CoV2^ completely abolished the remodeling, while swapping motif 2 or motif 3 resulted in partial defects (Fig. 3d). Motif 2 swapping resulted in decreased expression (Fig. 3d, immunoblotting), although the level was still above what was sufficient to cause robust 3DB formation in 3a^CoV2^. Swapping motif 1, 2, or 3 individually in 3a^CoV1^ was not sufficient to restore 3DB formation (**Extended Data Fig. 5c**). These results indicate that multiple residues spanning all three motifs are important.

### The remodeling activity is conserved in ORF3a from multiple but not all SARSr-CoVs

**Figure 4:**
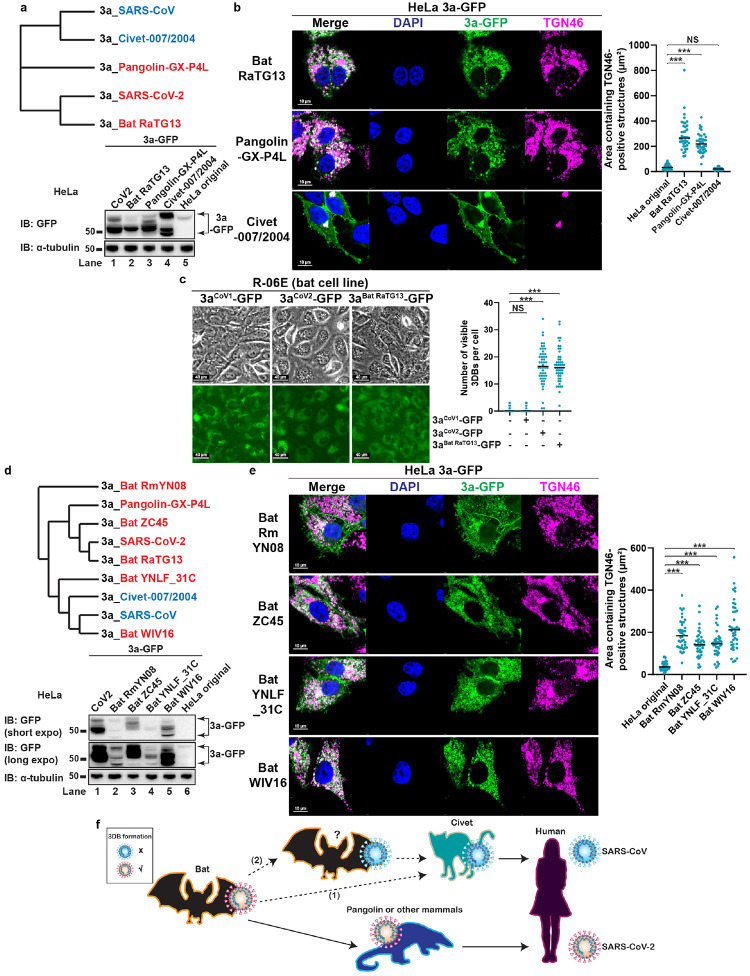
ORF3a-mediated 3DB assembly is conserved in multiple but not all SARSr-CoVs. (a) Phylogenetic tree of ORF3a proteins was constructed with Clustal Omega. ORF3a proteins with or without 3DB formation activity were labeled in red and blue, respectively. GFP immunoblotting was performed to compare the expression levels of 3a-GFP proteins. (b) HeLa cells stably expressing the indicated 3a-GFP proteins were immunostained for TGN46. Areas containing TGN46-positive structures were measured with ImageJ (n = 40 cells/sample; mean ± s.d.; two-sided t-test; ***, p<0.001; NS, not significant; black line indicates median value). Representative data from at least three independent experiments are shown. (c) The bat cell line R-06E was transduced with lentivirus to stably express the indicated 3a-GFP proteins.3DB formation was examined with phase contrast microscopy while the 3a-GFP levels were examined with fluorescence microscopy. The number of 3DBs per cell (visible on the current focal plane with clear DAPI signal) was quantified (n = 40 cells/sample; mean ± s.d.; two-sided t-test; ***, p<0.001; NS, not significant; black line indicates median value). Representative data from two independent experiments are shown. (d–e) Similar to (a–b), except four additional bat SARSr-CoV ORF3a proteins were examined. Representative data from at least three independent experiments are shown. (f) Model: two possible routes for the loss of 3DB formation activity during evolution to SARS-CoV: (1) ORF3a proteins in all bat SARSr-CoVs possess the activity, but the activity was lost during/after spillover from bat to civet; or (2) the 3DB formation activity was lost in a yet unidentified bat SARSr-CoV that is more closely related to SARS-CoV than Bat-CoV-WIV16. Note: the intermediate host for SARS-CoV-2 has not been fully confirmed, and therefore it is labeled as ‘pangolin or other mammals’.

To test whether 3DB formation is conserved in other SARSr-CoVs, we examined ORF3a derived from three SARSr-CoVs using the individual expression system in HeLa (Fig. 4a): (1) Bat-CoV-RaTG13, a horseshoe bat coronavirus that is one of the closest related coronaviruses to SARS-CoV-2^3^; (2) Pangolin-CoV-GX-P4L, a pangolin SARSr-CoV evolutionarily close to SARS-CoV-2^42–44^; (3) Civet-CoV-007/2004, a civet SARSr-CoV proposed to be the intermediate species for SARS-CoV^45^. Consistent with their evolutionary distance with SARS-CoV-2 and SARS-CoV, the bat and pangolin ORF3a induced profound 3DB formation, while the civet ORF3a behaved similarly to 3a^CoV1^ (Fig. 4b).

The observation that 3a^Bat RaTG13^ induced robust 3DB formation raised the question as to whether this remodeling activity occurs in bats, the host organisms for progenitor coronaviruses of both SARS-CoV-2 and SARS-CoV^10^. We adapted the individual expression system to R-06E, an Egyptian fruit bat (*Rousettus aegyptiacus*) embryonal cell line^46^. The *Rousettus aegyptiacus* TGN46 protein sequence is significantly different from the human one and thus cannot be recognized by immunostaining. Instead, we used the phase contrast microscopy method to detect 3DB formation. A large number of 3DBs were formed in R-06E cells expressing 3a^CoV2^-GFP or 3a^Bat RaTG13^-GFP, but not in those expressing 3a^CoV1^-GFP (Fig. 4c). Our results confirm that the cellular mechanisms supporting 3DB formation is conserved in bat cells.

The absence of 3DB formation in 3a^Civet-CoV−007/2004^ made us wonder whether the watershed event for ORF3a to acquire or lose this activity preceded the spillovers from bats to other animal hosts. To answer this question, we characterized four additional bat SARSr-CoV ORF3a homologs (Fig. 4d) in HeLa, chosen based on varied evolutionary distance to 3a^CoV1^ and 3a^CoV2^. These bat ORF3a proteins were expressed at varied levels and all of them were lower than 3a^CoV2^ (Fig. 4d), probably due to the suboptimal adaptation to human codons. Nevertheless, all four bat ORF3a promoted robust 3DB formation (Fig. 4e). Unexpectedly, this included ORF3a from Bat-CoV-WIV16, a close relative to SARS-CoV^47^. These results suggest that 3DB formation is highly conserved in bat SARSr-CoVs. However, this activity was lost either (1) during/after spillover from bat to civet, or (2) in a yet unidentified bat SARSr-CoV that is more closely related to SARS-CoV than Bat-CoV-WIV16 (Fig. 4f).

### S171 and W193 are key residues for 3DB formation

We have now identified two distinct groups of ORF3a based on whether they possess (Group I) or lack (Group II) the ability to form 3DBs (**Extended Data Fig. 6a**). Interestingly, alignment of motif 1–3 revealed that motif 3 sequences (orange residues) are 100% identical in Group II ORF3a and 3a^Bat WIV16^, suggesting that while motif 3 is important for maintaining high remodeling activity in 3a^CoV2^, other motifs can support 3DB formation in 3a^Bat WIV16^. We noticed that aa E171 and R193, located in motif 1 and motif 2, respectively, are the only two residues that exclusively appear in Group II but not Group I ORF3a, suggesting that these two residues may be important in defining the difference. Consistent with this hypothesis, swapping aa 171 in 3a^CoV2^ to that of 3a^CoV1^ (S171E) completely abolished 3DB formation, while swapping aa 193 (W193R) partially reduced the activity (**Extended Data Fig. 6b**). As expected, swapping both residues (S171E/W193R) caused complete defect similar to S171E (**Extended Data Fig. 6b**). Swapping of aa 171 and 193 in 3a^CoV1^ at the same time (E171S/R193W), but not individually (E171S or R193W), restored 3DB formation (**Extended Data Fig. 6c**). These results indicate that aa 171 in motif 1 and aa 193 in motif 2 are both important and work together to support the remodeling. It also explains why swapping motif 1 and motif 2 individually in 3a^CoV1^ did not restore 3DB formation (**Extended Data Fig. 5c**), as swapping both is essential for restoring the activity.

### Engineering of a recombinant SARS-CoV-2 mutant defective in 3DB formation

We aimed to engineer a SARS-CoV-2 mutant virus specifically defective in 3DB assembly to investigate its functions during viral infection. Because motif 3 only contains five residues (aa 209, 210, 215, 219, and 220) different between 3a^CoV2^ and 3a^CoV1^, we designed a mutant with these five residues plus aa 171 and 193 swapped with 3a^CoV1^ (3a^CoV2 7 aa swap^) to disrupt any residual remodeling activity. 3a^CoV2 7 aa swap^ had complete defect in 3DB formation, while still retaining strong expression and localization pattern similar to 3a^CoV1^ (**Extended Data Fig. 6c**). Because SARS-CoV/SARS-CoV-2 chimeric viruses are classified as select agents by the Centers for Disease Control and Prevention (CDC)^48^ due to concerns of potential gain of functions, we designed another 3a^CoV2^ mutant with these seven residues mutated to alanine (3a^CoV2_7Ala^) (**Extended Data Fig. 6d**). Similar to 3a^CoV2 7 aa swap^, 3a^CoV2_7Ala^ was expressed at comparable level to 3a^CoV2^, shared similar localization pattern with 3a^CoV1^, and exhibited a significant defect in 3DB formation (**Extended Data Fig. 6e–g**). Consistently, the giant 3DB structures under TEM disappeared in cells expressing this mutant (**Extended Data Fig. 3e**).

**Figure 5:**
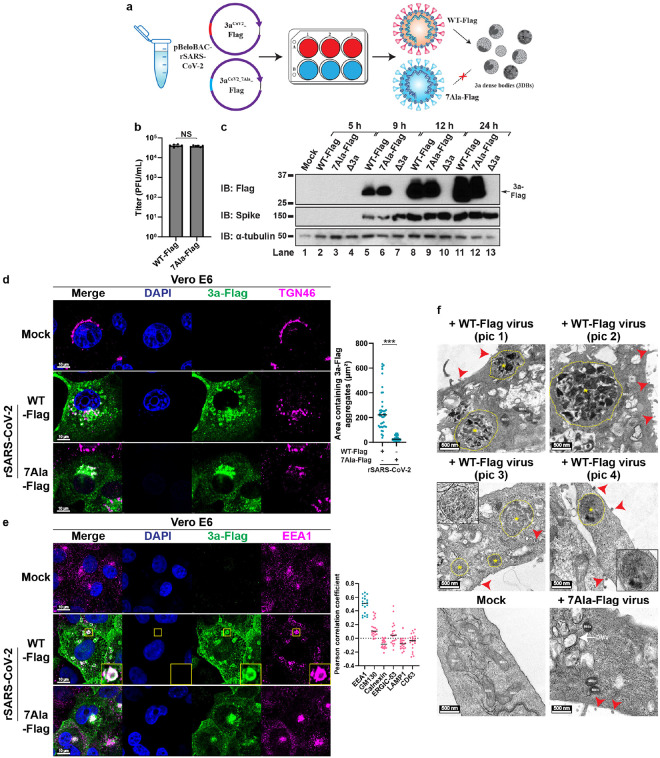
3DBs are assembled during SARS-CoV-2 infection. (a) Schematic for generation of recombinant SARS-CoV-2 (rSARS-CoV-2). The bacterial artificial chromosome (BAC) plasmid pBeloBAC11 was engineered to encode the viral genome of SARS-CoV-2. The ORF3a gene was replaced by either 3aCoV2(WT)-Flag or 3aCoV2_7Ala-Flag. Vero E6 cells were transfected with the BAC plasmids before virus-containing supernatants were collected and propagated to generate viral stocks for WT-Flag and 7Ala-Flag virus. WT-Flag but not 7Ala-Flag virus can form 3DBs. (b) Vero E6 cells were infected with the indicated rSARS-CoV-2 viruses in serial dilutions for 72 hours (h) for plaque assay. The plaque numbers were counted to calculate the titers in plaque-forming unit (PFU). Mean ± s.d.; two-sided t-test; NS, not significant. Data from three independent experiments (representative from at least six independent experiments) are shown, each with duplicate plates, and normalized to the total titers of the first experiment. (c) Vero E6 cells were infected with rSARS-CoV-2 viruses at an MOI of 0.1 for the indicated time. The cells were then lysed in RIPA buffer for immunoblotting. Spike = spike S2 antibody. Representative data from at least three independent experiments are shown. (d) Vero E6 cells were infected with the indicated rSARS-CoV-2 viruses at an MOI of 0.1 for 24 h, before immunostained for Flag and TGN46. Areas containing 3a-Flag-positive structures (3DBs in WT-Flag virusinfected cells or cluster in 7Ala-Flag virus-infected cells) were measured with ImageJ (n = 40 cells/sample; mean ± s.d.; two-sided t-test; ***, p<0.001; black line indicates median value). Representative data from at least three independent experiments are shown. (e) Vero E6 cells were infected as in (d) and immunostained for Flag and EEA1. For Vero E6 cells infected with WT-Flag virus, colocalization of 3DBs with different organelle markers (images in Fig. 6e and Extended Data Fig. 8b) were quantified with Pearson correlation coefficient using Coloc 2 plugin of ImageJ (n = 20 cells/sample; threshold regression: Costes). Organelle makers with strong and weak colocalization with 3DBs are labeled in blue and pink, respectively. Representative data from two independent experiments are shown. (f) Vero E6 cells were infected as in (d) and imaged with TEM. Four pictures (pic 1–4) are shown to highlight different subtypes of 3DBs (labeled with * and yellow outline; pic 1 and 2 show type (i) 3DBs; pic 3 and 4 show type (ii) and (iii) 3DBs). 3DBs only appeared in cells infected with WT-Flag virus. Insets: higher magnification of two 3DBs. Mito, mitochondria; Nu, nucleus; DMV, double-membrane vesicle. Red arrowheads indicate several of the virions. Representative images from two biological repeats (>40 cells per condition) are shown.

Using a bacterial-artificial-chromosome (BAC)-based reverse genetic system^49–51^, we engineered two recombinant SARS-CoV-2 (rSARS-CoV-2) viruses based on the genomic sequence of USA-WA1 strain: one with a Flag-tag inserted at the C-terminus of WT 3a^CoV2^ (referred to as WT-Flag virus), and the other with 3a^CoV2^ replaced by 3a^CoV2_7Ala^ with a C-terminal Flag-tag (referred to as 7Ala-Flag virus) (Fig. 5a). The C-terminal Flag-tag was added to allow immunoblotting and immunostaining of ORF3a. We confirmed that both viruses contained the intended genomic sequences using next-generation sequencing technology (see Methods), and that WT-Flag virus propagated similarly to a previously characterized rSARS-CoV-2 virus without a Flag-tag^49–51^. Both WT-Flag and 7Ala-Flag viruses showed comparable titers in plaque assays (Fig. 5b). When infecting Vero E6 cells, both viruses produced strong and comparable amounts of intracellular viral proteins including ORF3a in a 24-hour (h) time course experiment (Fig. 5c). These data suggest that 3DB formation is not essential for viral protein synthesis or production of infectious virions, consistent with our previous finding that Δ3a virus did not show significant defect in viral titers^15^.

### 3DBs are loaded with viral spike (S) and membrane (M)

Previous studies have shown that SARS-CoV-2 infection leads to a complete fragmentation of the Golgi apparatus, including the *cis*-Golgi^4,31^. The Golgi fragmentation was proposed to be induced by multiple viral factors other than 3a^CoV2 52^. Consistent with these studies, we observed that (1) SARS-CoV-2 induced dramatic dispersion of TGN46-positive structures (**Extended Data Fig. 7a–b**), but the effect was not dependent on the presence of ORF3a (**Extended Data Fig. 7c**); (2) SARS-CoV-2 infection also induced the fragmentation of the *cis*-Golgi (**Extended Data Fig. 7d**), in contrast to the lack of effect on the *cis*-Golgi morphology by 3a^CoV2^ in the individual expression system. Therefore, dispersion of TGN46-positive structures is not a suitable hallmark for studying 3a^CoV2^-mediated remodeling during viral infection due to the interference of other viral factors. Instead, we focused on monitoring 3DBs via Flag immunostaining. Vero E6 cells were infected with WT-Flag or 7Ala-Flag virus at a multiplicity of infection (MOI) of 0.1 and imaged at 24 h post-infection (hpi). As shown in Fig. 5d, WT-Flag virus infection led to the formation of multiple giant 3DBs (1.81 ± 0.65 μm in diameter) positive with 3a^CoV2^-Flag. In contrast, in 7Ala-Flag virus-infected cells, the formation of 3DBs was abolished, while 3a^CoV2_7Ala^-Flag was enriched on a perinuclear cluster (Fig. 5d), recapitulating the localization of this mutant in the individual expression system. Similar results were also observed in A549-hACE2 cells (**Extended Data Fig. 8a**). These results indicate that 3a^CoV2^ drives 3DB formation during viral infection in a way dependent on the seven key residues.

Colocalization between 3DBs and TGN46 was observed in infected cells, but less prominent than the individual expression system, probably due to the additional TGN fragmentation caused by other viral factors. Consistent with the individual expression system, 3DBs formed during infection were not positive with organelle markers of the *cis*-Golgi, ER, ERGIC, or lysosome (**Extended Data Fig. 8b**, quantification in Fig. 5e). CD63, a marker of MVBs, exosomes, late endosomes, and lysosomes^53,54^, was not detected on 3DBs either (**Extended Data Fig. 8b**, quantification in Fig. 5e). In contrast, the early endosome marker EEA1 was highly enriched on 3DBs (Fig. 5e). These results again support the TGN and early endosomal origin of 3DBs. When imaged with TEM, the electron-dense 3DBs were only detected in cells infected with WT-Flag virus, but not those infected with 7Ala-Flag virus (Fig. 5f). More than 90% of WT-Flag virus-infected cells (n>80 cells in two biological repeats) showed at least one 3DB in the current cut section. In contrast, DMVs, intracellular virions, and budding virions, were detected for both viruses (Fig. 5f and data not shown). We also observed that the number of 3DBs, as detected by both fluorescence microscopy and TEM, was lower during infection than the individual expression system (see Discussion).

**Figure 6:**
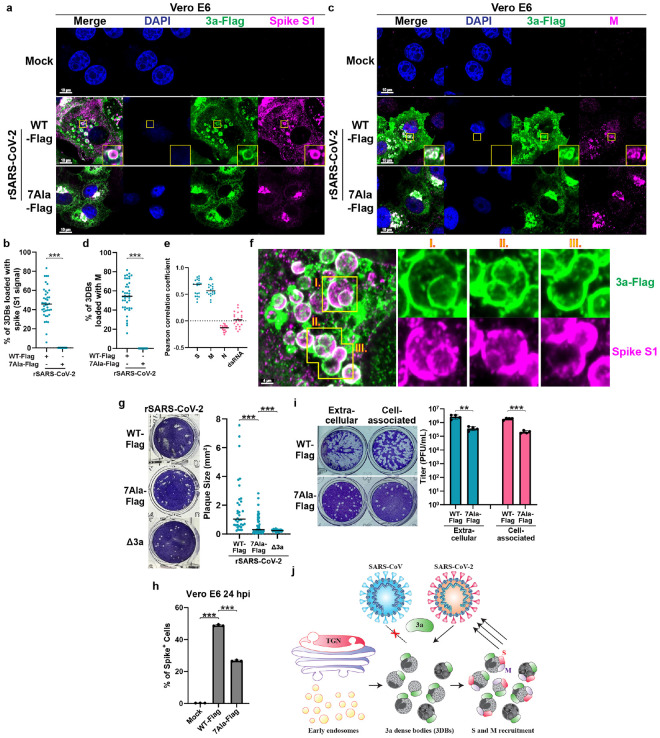
3DBs are loaded with S and M and are essential for optimal viral infectivity. (a–b) Vero E6 cells were infected with the indicated rSARS-CoV-2 viruses at an MOI of 0.1 for 24 h, before immunostained for Flag and spike S1. The percentage of 3DBs loaded with spike was quantified (n = 40 infected cells/sample; mean ± s.d.; two-sided t-test; ***, p<0.001; black line indicates median value). Representative data from at least three independent experiments are shown. (c–d) Similar to (a-b), except the cells were immunostained for Flag and membrane (M). The percentage of 3DBs loaded with M was quantified with methods similar to (a–b). (e) For Vero E6 cells infected with WT-Flag virus, colocalization of 3DBs with S, M, N, or dsRNA (images in Fig. 6a, 6c, and Extended Data Fig. 9c–d) were quantified with Pearson correlation coefficient using Coloc 2 plugin of ImageJ (n = 20 cells/sample; threshold regression: Costes). Strong and weak colocalization with 3DBs are labeled in blue and pink, respectively. Representative data from at least three independent experiments are shown. (f) A magnified image of Vero E6 infected with WT-Flag virus and immunostained for Flag and spike S1 as in (a) is shown. Three regions were highlighted: I. and II. show large 3DBs containing smaller 3DBs; III. shows a fusion or fission event between 3DBs. Representative data from at least three independent experiments are shown. (g) Vero E6 cells were infected with the indicated rSARS-CoV-2 viruses for 72 h for plaque assay with 1.25% carboxymethylcellulose overlay medium. The plaque size was measured with ImageJ (duplicate plates per sample; mean ± s.d.; two-sided t-test; ***, p<0.001; black line indicates median value). Representative data from at least six independent experiments are shown. (h) Vero E6 cells were infected with the indicated rSARS-CoV-2 viruses at an MOI of 0.1 for 24 h. The cells were fixed and stained for spike S2 antibody followed by Alexa Fluor 568. The cells were then sorted by flow cytometry to quantify spike-positive cells (triplicate measurement; mean ± s.d.; two-sided t-test; ***, p<0.001). Representative data from at least four independent experiments are shown. (i) Vero E6 cells were infected with the indicated rSARS-CoV-2 viruses at an MOI of 0.1 for 1 h before washed and incubated in fresh medium. After another 23 h (for a total of 24-h infection), the medium and lysate were collected for plaque assay to determine the extracellular and cell-associated viral titers, respectively. Representative plaque assay images are shown for the same titration (all wells were from the same plate; plaque numbers for WT-Flag virus were measured from further diluted wells not shown here). Mean ± s.d.; two-sided t-test; **, p<0.01; ***, p<0.001. Representative results from at least four independent experiments are shown. (j) Model: ORF3a from SARS-CoV-2 but not SARS-CoV hijacks a specific subset of TGN and early endosomal membranes to build giant dynamic electron-dense 3DBs. 3DBs are loaded with the viral structural proteins S and M to facilitate their trafficking for virion assembly. 3DB formation is essential for SARS-CoV-2 to achieve maximal viral infectivity.

During virion assembly, the viral structural protein S is incorporated into the viral lipid envelope and is responsible for binding to ACE2 receptor on host cells to mediate viral entry^55^. Notably, 3DBs were loaded with S, as confirmed by immunostaining with two antibodies recognizing the S1 subunit and S2 subunit of S, respectively, in both Vero E6 and A549-hACE2 cells (Fig. 6a–b and **Extended Data Fig. 9a–b**). While all S-positive spherical structures had 3a^CoV2^ signal, only a subset of 3DBs were loaded with S. In addition, in 7Ala-Flag virus-infected cells, the giant spherical structural localization of S disappeared (Fig. 6a–b and **Extended Data Fig. 9a–b**). These results suggest that 3a^CoV2^ forms 3DBs to recruit S. Another viral structural protein M is also incorporated into the viral lipid envelope and serves as a scaffold for virion assembly^56^. We found that M was also recruited to 3DBs in a manner dependent on the seven key residues of 3a^CoV2^ (Fig. 6c–d). In contrast, nucleocapsid (N), a viral structural protein that encapsulates the viral RNA^57^, did not localize to 3DBs (**Extended Data Fig. 9c**, quantification in Fig. 6e). This is consistent with a previous study showing that N shares limited colocalization with other structural proteins, including S and M, indicating that N uses a different trafficking route for virion assembly^31^. Double-stranded RNA (dsRNA), a product of SARS-CoV-2 viral genome replication and mRNA transcription^58^, was not detected on 3DBs either (**Extended Data Fig. 9d**, quantification in Fig. 6e). The lack of N, dsRNA, and ER/ERGIC markers on 3DBs suggests that 3DBs are distinct from the previously characterized replication organelles. We propose that 3DBs are membrane structures specifically involved in the trafficking of S and M for virion assembly. Consistent with this hypothesis, 3DBs underwent constant fusion and/or fission events, as well as engulfment of smaller 3DBs (Fig. 6f). These observations suggest that 3DBs are highly dynamic and constantly exchange the loaded cargos S and M. While 3DBs were deprived of N, dsRNA, ER marker and ERGIC marker, 3DBs were in proximity to these structures, suggesting that 3DBs may be highly interconnected with the replication organelles to facilitate viral protein trafficking and virion assembly.

To study the kinetics of 3DBs formation, we imaged infected Vero E6 cells at an MOI of 0.1 for 5, 8, and 15 h. These time points were chosen to represent the three stages of viral protein expression^31^. Both WT 3a^CoV2^ and 3a^CoV2_7Ala^ became detectable in a small subset (~1%) of infected cells at 5 hpi, before S and M became detectable (**Extended Data Fig. 10a–b**). At this early time point, WT 3a^CoV2^ was localized on tubular and punctate structures. At 8 hpi, WT 3a^CoV2^ formed small 3DBs that were clustered together, which recruited S and M (**Extended Data Fig. 10a–b**). At 15 hpi, 3DBs became larger. In contrast, while N became detectable as early as 5 hpi, it did not localize on 3DBs during the entire time course (**Extended Data Fig. 10c**). These data indicate that 3a^CoV2^ is one of the early synthesized viral proteins and form 3DBs between 5 and 8 hpi. The growth in 3DB size may be a result of the constant fusion.

### 3DBs are required for maximal viral infectivity

We previously showed that rSARS-CoV-2 Δ3a produced reduced plaque size^15^, suggesting that 3a^CoV2^ is important for optimal viral infectivity. To study the contributions of 3DB formation, we compared the plaque size of WT-Flag virus, 7Ala-Flag virus, and Δ3a virus. 7Ala-Flag virus consistently showed significantly smaller plaques compared to WT-Flag virus, although the average plaque size was still larger than that of Δ3a virus (Fig. 6g). Our data suggest that 3a^CoV2^ possesses both 3DB-dependent and -independent functions to facilitate viral spread (see Discussion).

Plaque assays can only quantify viral spread starting from day 3 post infection due to the small plaque size in the first two days. To examine viral spread in the first 24 h, we infected Vero E6 with WT-Flag virus and 7Ala-Flag virus at an MOI of 0.1. At 24 hpi, the cells were fixed and stained with S antibody followed by Alexa Fluor 568, before analyzed by flow cytometry to quantify the percentage of infected cells (spike^+^). The populations were analyzed by size and morphology to ensure that no significant cytotoxicity occurred at this time point. To exclude effects caused by titer decrease during storage or freezing/thawing, we re-measured the titers at the same time to confirm that the two viruses were maintained at the same titer. 7Ala-Flag virus consistently infected a lower percentage (~50% decrease) of cells compared to WT-Flag virus at 24 hpi (Fig. 6h).

Next, we examined the infectivity of both extracellular (i.e., virions released into the medium) and cell-associated (i.e., both intracellular and cell-bound virions) virus. Vero E6 was infected with WT-Flag virus and 7Ala-Flag virus at an MOI of 0.1 for 1 h, before the medium was removed and the cells were washed with PBS. The cells were then incubated in fresh medium for another 23 h for a total of 24-h infection. The supernatant and cell lysate were then collected separately for plaque assay to measure the extracellular and cell-associated viral titer, respectively. For both cases, 7Ala-Flag virus consistently showed ~10-fold reduction in viral titers (Fig. 6i). Together with the observation that 7Ala-Flag virus infection showed comparable level of intracellular viral proteins with WT-Flag virus infection (Fig. 5c), our data suggest that 3DBs are critical for efficient virion assembly to achieve optimal infectivity. In contrast, the defect was not further amplified in the extracellular virus, suggesting that viral egress may not be affected by the lack of 3DB formation. While the measurement of spike^+^ cells in Fig. 6h reflects a snapshot of infection efficiency at 24 hpi, the measurement of extracellular and cell-associated viral titers reflects the capacity of virus to continue infecting cells beyond 24 h. The enhanced defect in the latter (~10-fold reduction in Fig. 6i vs. ~50% decrease in Fig. 6h) indicates that the contributions of 3DBs for infectivity increase as infection progresses. Together, our findings indicate that 3a^CoV2^ hijacks a specific subset of the host TGN and early endosomal membranes to form giant dense bodies, which facilitates the trafficking of S and M for optimal infectivity of SARS-CoV-2 (Fig. 6j).

## DISCUSSION

Here we identified and characterized 3DBs, 3a^CoV2^-driven membrane structures assembled during SARS-CoV-2 infection. The unusual electron-dense nature and membranous sub-compartments of 3DBs distinguish them from other organelles such as nigericin-induced TGN vesicles, DMVs, and MVBs. Electron-dense nature with TEM is usually correlated with a large amount of protein and lipids, but can also indicate the presence of metal elements, phosphate, or other chemicals^59,60^. In addition, 3DBs show several different morphologies ((i)– (v) in Fig. 1c). In both individual system and infection system, (i) and (ii) were the most abundant forms, indicating that they may be the mature or most stable forms. The membranous sub-compartments observed in these two forms may be related to the small 3DBs engulfed in giant 3DBs (Fig. 6f). In contrast, (iv) and (v), the most electron-dense 3DB structures, were relatively smaller than the other three forms in the individual expression system (Fig. 1c), and rarely detectable in the infection system at 24 hpi, suggesting that they may represent either the early stages or the less stable end stages of 3DBs.

In the individual expression system, TGN46 was abundantly localized on most (if not all) 3DBs while EEA1 was only recruited to a subset of 3DBs (Fig. 2b). In contrast, during SARS-CoV-2 infection, EEA1 was recruited to a large amount of 3DBs (Fig. 5e) while TGN46 was only detected on a subset of 3DBs (Fig. 5d). These differences suggest that other viral factors may have additional effects on the membrane remodeling. For example, Golgi fragmentation may reduce the amount of TGN membrane available for 3DB formation. These observations also indicate that the recruitment of TGN46 and EEA1 is not entirely dependent on each other as they have different recruitment patterns to 3DBs, although we cannot exclude the possible involvement of cargo exchange between the TGN and early endosomes. SARS-CoV-2 uses the Golgi apparatus for virion trafficking and post-translational modifications^61^. Therefore, one possibility why 3a^CoV2^ targets a narrow range of host TGN membrane may be to prevent interfering with the Golgi apparatus hijacking by other viral factors. This highlights the complexity and well-coordinated nature of virus-mediated host organelle remodeling.

Another difference between the infection system and the individual expression system is that the number of 3DBs formed during infection is lower while the diameter is higher (e.g., Fig. 5d vs. Extended Data Fig. 3a, both in Vero E6). This was not due to overexpression in the individual expression system, as individually expressed 3a^CoV2^ induced a large number of small 3DBs even at a barely detectable level (**Extended Data Fig. 3c**). One possibility is that the complete Golgi fragmentation by other viral factors reduced the amount of TGN membrane 3a^CoV2^ can hijack during infection, resulting in lower number of 3DBs. The increase in 3DB diameter during infection may be caused by constant fusion and/or fission events (Fig. 6f). Indeed, when 3DBs first appeared at 8 hpi, they were small structures resembling those in the individual expression system, before growing larger at 15 hpi (**Extended Data Fig. 10**). The growth in size may be facilitated by the loading of S and M, although other viral factors may also regulate 3DB size. One interesting observation is that 3a^CoV2_7Ala^ localized on a perinuclear cluster structure during infection (Fig. 5d). This perinuclear cluster resembled the Golgi apparatus and was located in proximity to the dispersed *cis*-Golgi marker GM130 (**Extended Data Fig. 8b**), thus raising the question whether part of the Golgi apparatus remains intact during infection. An extensive characterization of Golgi markers during SARS-CoV-2 infection may help answer this question.

Consistent with our previous discovery that the dispersion of PtdIns4P-positive TGN structure is required for the NLRP3 inflammasome assembly and activation^29^, neither 3a^CoV1^ nor 3a^CoV2^ activates the NLRP3 inflammasome (**Extended Data Fig. 4c–d**). This is in contrast to other studies proposing that both 3a^CoV1^ and 3a^CoV2^ activate the NLRP3 inflammasome^62–64^. The discrepancies may be due to different cell models and expression systems used. While we observed minimal inflammasome activation in a RAW 264.7 infection model (**Extended Data Fig. 4e**), we cannot exclude the possibility that SARS-CoV-2 may activate the NLRP3 inflammasome in other cell types or *in vivo* as indicated by other studies^65–67^.

ORF3a homologs in bat and pangolin coronaviruses also have the 3DB formation activity (Fig. 4b). Unexpectedly, this activity was lost in ORF3a from SARS-CoV and a closely related civet coronavirus (Fig. 1a and 4b). Further characterization of the remodeling activity in other bat and animal SARSr-CoVs will provide important insights into the divergence of coronaviruses that lead to the evolution of SARS-CoV. While both SARS-CoV and SARS-CoV-2 are highly similar in genome sequence (79% genome sequence identity)^27^, they differ greatly in transmission rates, pathogenesis and host immune responses^68^. Our discovery that ORF3a in these two viruses possess strikingly different ability to assemble 3DBs provides a new direction to understand the different features of these two coronaviruses, especially for the highly contagious nature of SARS-CoV-2. Bat coronaviruses serve as reservoirs for a number of important emerging HCoVs. Therefore, close genomic monitoring of bat coronaviruses for changes in 3DB formation activity will provide insights into identifying future pathogenic HCoVs with pandemic potential.

One of the major questions remained to be answered is how 3a^CoV2^ hijacks host membranes to form these giant dense bodies. While the viroporin activity of 3a^CoV2^ has been supported by a previous structural study^14^, a recent study has suggested that 3a^CoV2^ is not a viroporin^13^. It thus remains to be determined whether the viroporin activity of 3a^CoV2^ exists, and if so, whether it is involved in 3DB assembly. Our immunoblotting results show that ORF3a proteins from SARSr-CoVs often appeared as multiple bands (e.g., Fig. 4a/4d), indicating that they may undergo extensive post-translational modifications (PTMs) or proteolytic cleavage. However, we did not observe strong correlations between protein band positions and 3DB formation activity. Therefore, whether the remodeling activity is dependent on particular PTMs or cleavage events still remains to be studied. Finally, it remains to be investigated whether host factors are essential to facilitate the 3DB assembly, or 3a^CoV2^ alone is sufficient to form these structures. We have demonstrated that a small Flag-tag can be inserted at the C-terminus of 3a^CoV2^ without disrupting virion assembly or viral propagation. This will allow future identification and characterization of 3a^CoV2^ PTMs and binding partners during infection using Flag immunoprecipitation coupled to mass spectrometry.

Coronaviruses possess the largest genomes in RNA viruses, and thus it is technically challenging and time-consuming to engineer recombinant SARS-CoV-2 mutants. We therefore took advantage of the individual expression system for domain swapping to identify the key motifs for 3DB formation. We successfully identified seven key residues in the C-term. This allowed us to engineer a mutant virus defective in 3DB formation. While the ability to form 3DBs does not affect the viral protein synthesis (Fig. 5c), it is required for optimal infectivity of both cell-associated and extracellular virions (Fig. 6i). Our findings that 3DBs are loaded with S and M (e.g., Fig. 6a/**6c**) indicate that S and M may use 3DBs as an enrichment route distinct from the DMVs used by N and dsRNA. It remains to be characterized how 3DBs promote higher efficiency for virion assembly, either by increasing the number of assembled virions or by assembling more infectious virions. It will be interesting to investigate whether 3a^CoV2^, S, and M form distinct structures or oligomers on 3DBs. While dramatically different in morphologies, organelle origin, and loaded viral components, 3DBs and DMVs share two common features: (1) DMVs appeared in infected cells at 6–8 hpi^4,69^, which overlaps with the time when 3DBs appeared (5–8 hpi) (**Extended Data Fig. 10**); (2) both DMVs^4^ and 3DBs (Fig. 6f) had contacts between individual structures that suggested fusion and/or fission, and they both had larger structures containing smaller ones. While future experiments are needed to explore their relationships, it is possible that these two types of virus-induced structures are closely interconnected to facilitate virion assembly. Our previous study has confirmed the critical role of 3a^CoV2^ in pathogenesis in a mouse model^15^. It remains to be determined the contributions of 3DBs in SARS-CoV-2 virulence *in vivo*.

Disruption of 3DB formation with 7Ala mutations still retained the colocalization of S and M with the 3a^CoV2_7Ala^ cluster (Fig. 6a and 6c), indicating that the cluster may still retain suboptimal function to facilitate virion assembly. This is consistent with the intermediate defects of 7Ala-Flag virus between WT-Flag virus and Δ3a virus (Fig. 6g). Alternatively, previous studies have shown that 3a^CoV2^ is involved in late endosome/lysosome trafficking and autophagy^13,16–25^, which may also account for the 3DB-independent function of 3a^CoV2^ on plaque size. This may explain why SARS-CoV maintains the 3a^CoV1^ gene despites its complete loss of 3DB formation activity.

Finally, while we focused on the reference strain SARS-CoV-2 USA-WA1 in this study, it will be interesting to investigate whether the key motifs and residues are mutated to the nonremodeling version in other variants. A recent study^70^ highlights a few unique ORF3a mutations in Omicron that are absent in other variants. However, none of these are in the aa 171–222 region, consistent with the importance of 3DB assembly driven by this region for viral transmission. Another study^71^ found that S171L mutation was found in ORF3a of some circulating strains. Whether this mutation disrupts the 3DB formation activity of 3a^CoV2^ and the resulting effects on pathogenicity remain to be determined.

In summary, we have identified ORF3a from SARS-CoV-2 and related coronaviruses as a specific type of membrane-remodeling viral factors to assemble dynamic electron-dense bodies, which are required for optimal viral infectivity. Our discovery will provide important insights into coronavirus cell biology and the development of COVID-19 prophylactics and therapeutics.

## Data Availability

All data supporting the findings of this study are available from the corresponding author.
